# DONALD P. KENT AND ROBERT W. KLEEMEIER AWARD LECTURES

**DOI:** 10.1093/geroni/igae098.1856

**Published:** 2024-12-31

**Authors:** Judith Howe

**Affiliations:** Chair

## Abstract

The Donald P. Kent Award lecture will feature an address by the 2023 Kent Award recipient Kathleen Wilber, PhD, FGSA, FAGHE of the University of Southern California. The Robert W. Kleemeier Award lecture will feature an address by the 2023 Kleemeier Award recipient David M. Almeida, PhD, FGSA of Pennsylvania State University. The Kent Award is given annually to a member of The Gerontological Society of America who best exemplifies the highest standards of professional leadership in gerontology through teaching, service, and interpretation of gerontology to the larger society. The Kleemeier Award is given annually to a member of The Gerontological Society of America in recognition for outstanding research in the field of gerontology.

